# Composition and Structure of Gut Microbiota of Wild and Captive *Epinephelus morio* via 16S rRNA Analysis and Functional Prediction

**DOI:** 10.3390/microorganisms13081792

**Published:** 2025-07-31

**Authors:** Grecia Montalvo-Fernández, Joanna M. Ortiz-Alcantara, Claudia Durruty-Lagunes, Laura Espinosa-Asuar, Mariela Beatriz Reyes-Sosa, María Leticia Arena-Ortiz

**Affiliations:** 1 Laboratorio de Estudios Ecogenómicos, Facultad de Ciencias, Universidad Nacional Autónoma de México (UNAM), Parque Científico Tecnológico de Yucatán, Carretera Sierra Papacal—Chuburná Puerto, Km, 5.5, Tablaje 31257, Sierra Papacal, Mérida 97302, Yucatán, Mexico; grecia.montalvo@enesmerida.unam.mx (G.M.-F.); joanna.ortiz@ciencias.unam.mx (J.M.O.-A.); 2 Unidad Multidisciplinaria de Docencia e Investigación (UMDI) Sisal, Facultad de Ciencias, Universidad Nacional Autónoma de México (UNAM), Puerto de Abrigo S/N, Sisal 97355, Yucatán, Mexico; cvdl@ciencias.unam.mx; 3 Instituto de Ecología, Universidad Nacional Autónoma de México (UNAM), Circuito Exterior S/N, Ciudad de México 04510, Mexico; lauasuar@ecologia.unam.mx; 4 Secretaría de Ciencia, Humanidades, Tecnología e Innovación (Secihti), Facultad de Ingeniería Química, Universidad Autónoma de Yucatán, Industrias No Contaminantes x Anillo Periférico Norte S/N, Mérida 97203, Yucatán, Mexico; mreyes@secihti.mx

**Keywords:** metabarcoding 16S rRNA, taxonomic diversity, gut microbiota, metabolic predictions, marine fish, red grouper

## Abstract

The gut microbiota plays an essential role in the host’s metabolism. Its composition and structure depend on biological and environmental factors. This work was designed to identify the composition and structure of the wild and captive red grouper (*Epinephelus morio*) microbiota and make predictions regarding its metabolic functions. Our hypothesis stated that wild and captive individuals would share the most abundant taxonomic groups, forming a core microbiota, and individuals in captivity might have exclusive taxonomic groups. Metagenomic DNA was extracted from the intestinal contents of wild and captive individuals. The 16S rRNA gene was amplified and sequenced using Illumina pair-end technology. QIIME2 pipeline was used for sequence analysis and alpha and beta diversity assessment. PICRUSt was used to infer metabolic functions. Twenty-nine phyla were identified; the most abundant were Pseudomonadota, Bacillota, Fusobacteriota, and Actinomycetota. The dominant genera were *Photobacterium*, *Vibrio*, *Cetobacterium*, and *Escherichia-Shigella*. The metabolic prediction analysis suggested that the *Epinephelus morio* gut microbiota is related to food digestion, the immune system, antioxidant enzymes, antibiotic resistance, and vitamin B12 transport. We concluded that the microbiota of *E. morio* established in captivity is sensitive to environmental changes such as water pollution, which can cause a decrease in diversity.

## 1. Introduction

The gut microbiota is a dynamic and complex system; it has been very exhaustively studied in humans and mammals. The expansion of aquaculture brought with it a rise in studies related to the microbiota in fish [1]. The microbiota plays a vital role in nutrition, food absorption, homeostasis, immunological protection, growth, and health [2,3]. Its composition depends on the physiological condition of the host, diet, and environmental factors such as water quality, temperature, pH, etc. [4].

The development of molecular techniques such as Next-Generation Sequencing (NGS) technologies, sequence databases, and advances in computational procedures allow us to analyze the biodiversity of microbial communities [5]. Illumina technology has been widely used for 16S rRNA gene sequencing of gastrointestinal tract microbiota of marine fish such as the brown-marbled grouper (*Epinephelus fuscoguttatus*), spotted coral grouper (*Plectropomus maculatus*), and Atlantic salmon (*Salmo salar*) [6]. 

Several studies have been carried out on the composition of fish microbiota. However, there are few published studies on the influence of captivity on the gut microbial community. The impact of captivity and natural habitats on gut microbiome in *Epinephelus akaara* during four different seasons was determined. Compared to wild *E. akaara*, the authors found a more complex microbial network in captive *E. akaara* as evidenced by the increased abundance of Bacillaceae, Moraxellaceae, and Enterobacteriaceae. In contrast, Vibrionaceae, Clostridiaceae, Flavobacteriaceae, and Rhodobacteraceae were found to be more active in wild *E. akaara*. Some core microorganisms, such as *Photobacterium* and Firmicutes, showed similar distribution patterns in both wild and captive groups. This study provides a relatively more comprehensive description of the gut microbiota in *E. akaara* and provides a valuable reference for its aquaculture [7].

The gut microbial community of wild and captive Malaysian mahseer (*Tor tambroides*) studied through 16S rRNA sequencing followed by functional prediction showed that the most abundant bacterial phyla in both wild and captive *T. tambroides* were Bacillota, Pseudomonadota, Fusobacteriota, and Bacteroidota. The wild specimens of *T. tambroides* contain a more diverse gut microbiota than the captive ones. *Cetobacterium* was found in wild and captive individuals, suggesting that it is part of the core microbiota of *T. tambroides.* The contribution of the intestinal microbiota was mainly associated with carbohydrate metabolism in captive individuals, while in wild individuals, there was a greater contribution to bile secretion and lysine biosynthesis. This study provided insight into the functions that the microbiota can perform for the benefit of the host [8].

A study was undertaken to examine the changes in the gut microbiota composition of wild Atlantic cod (*Gadus morhua*) consequential to captivity. The study revealed that the counts of intestinal microbes in wild-caught fish were not affected by captive rearing for six weeks. However, the diversity of the intestinal bacterial community was reduced in response to artificial feeding [9].

The probiotic function of bacteria in the intestinal tract of fish has been widely recognized for its ability to improve host health and performance. These beneficial bacteria can inhibit the growth of pathogenic microorganisms by competing for nutrients and space. They also produce many enzymes such as amylases by *Aeromonas* spp., *Bacillus subtilis*, Bacteridaceae, *Clostridium* spp., *Lactobacillus plantarum*, and *Staphylococcus* sp. and proteases and cellulases by *B. subtilis*, *L. plantarum*, and *Staphylococcus* sp. [10]. Inferring the possible functions of the *E. morio* microbiota will allow us to know whether it has potential as a probiotic and may open up a line of research on the isolation of these beneficial bacteria from the *E. morio* gut for long-term use in aquaculture.

Our study focused on the gut microbiota of the red grouper (*Epinephelus morio*) and is the first study examining both wild and captive specimens. There is a lack of information regarding the microbiota present in the gut contents of this fish and the potential functions that these microorganisms perform. It is also unknown whether the captive lifestyle alters the composition and structure of the microbiota compared to that of wild individuals. Our hypothesis stated that wild and captive individuals would share the most abundant taxonomic groups, forming a core microbiota, and individuals in captivity might have exclusive taxonomic groups, probably related to adaptation to captive conditions. Regarding the prediction of metabolic functions, we predicted that functions related to food absorption and digestion and to the stress conditions resulting from captivity could be identified. To address this hypothesis, we aimed to identify the gut microbiota associated with the gut contents of wild and captive red grouper using 16S rRNA metabarcoding. To further characterize it, we predicted the metabolic functions of the gut microbiota.

## 2. Materials and Methods

### 2.1. Fish and Culture Conditions

The wild fish used in this study were collected in July 2019 on the northern coast of Yucatan. During sampling, the sea surface temperature ranged between 27 and 29 °C and salinity ranged between 37 and 38 ppm. The fish were transferred to Unidad Multidisciplinaria de Docencia e Investigación (UMDI), Sisal, Facultad de Ciencias, UNAM, where they remained for a few days before the first sampling (2019). Subsequently, the groupers were kept in captivity, and annual sampling was conducted until 2024. The fish were kept in tanks with a capacity of 20 m^3^ and a depth of 90 m with a recirculation and thermal control system within the facilities of the UMDI Sisal UNAM. The experimental tanks received their water directly from a seawater-fed reservoir. Water quality in the experimental tanks was evaluated daily by measuring pH, salinity, dissolved oxygen, and temperature with a YSI ProQuatro Multiparameter [YSI, Yellow Springs, OH, USA]. Fish were fed semi-moist food made from fish meal and squid every third day to satiety.

### 2.2. Sample Collection and DNA Extraction

Adult fish kept in captivity were used; the average weight of the fish for each year of sampling was as follows: 2019 (3.45 g), 2020 (4.05 g), 2021 (4.00 g), 2022 (4.67 g), 2023 (5.02 g), and 2024 (3.64 g). In detail, the sample size (fish) for each of the experimental groups was as follows: wild individuals (2019), 23 samples; captive individuals in 2020, 20 samples; captive individuals in 2021, 6 samples; captive individuals in 2022, 5 samples; captive individuals in 2023, 6 samples; and captive individuals in 2024, 8 samples). Only one sample was collected from each fish. The intestinal content samples were processed independently. All captive individuals (2020–2024) were collected from the wild in 2019, but the same individuals were not always sampled. This was due to two issues: (1) we had mortality associated with the conditions in captivity, so the same individuals could not always be sampled, and (2) for ethical reasons, we avoided manipulating the same individuals as this generated stress. A noninvasive methodology was used to collect intestinal content samples to ensure animal welfare without causing death. The fish were anesthetized with 1 mL of clove oil per 20 L of water, and a probe was inserted into the cloaca to take a sample of the intestinal contents. Each probe was placed in a sterile 1.5 mL Eppendorf tube, transported in liquid nitrogen to the laboratory, and stored at −80 °C. In the first year (2019), samples were taken from wild individuals, and subsequently, annual samples were taken from captive individuals between 2020 and 2024. The probes containing the intestinal contents were manually fragmented with sterile scissors, and 750 µL of lysis buffer was added to each tube. They were incubated for 10 min with agitation to extract the intestinal contents. Metagenomic DNA was extracted using the ZymoBIOMICS Kit [Zymo Research, Irvine, CA, USA]. DNA was quantified in a Quantus Fluorometer [Promega Corporation, Madison, WI, USA], and its integrity was determined in a 1% agarose gel.

### 2.3. Amplification, Sequencing of 16S rRNA Gene, and Sequence Processing

The 16S rRNA metabarcoding approach was used to characterize the gut microbiota. The V3–V4 hypervariable region was amplified with barcoded forward and reverse primers according to the protocol recommended by Illumina. The primer information can be seen in the Supplementary Materials (Supplementary File S1). The Phusion Flash High-Fidelity PCR master mix [Thermo Fisher Scientific, Waltham, MA, USA] was used in reaction volumes of 20 μL. Amplification was performed in a T100 thermal cycler [Bio-Rad, Hercules, CA, USA] conditions were as follows: 95 °C for 3 min, 25 cycles at 95 °C for 30 s, 55 °C for 30 s, 72 °C for 30 s, and a final extension at 72 °C for 5 min. Finally, 16S amplicons were quantified in a Qubit fluorometer [Thermo Fisher Scientific, Waltham, MA, USA] and sequenced within the Illumina Next-Seq 500 platform [Illumina, San Diego, CA, USA] (2 × 150 PE) using forward and reverse reads. The sequences were analyzed using Quantitative Insights into Microbial Ecology (QIIME2 ver. 2022.2) [11]. The q2-dada2 de-noise-paired algorithm was used. Sequences were demultiplexed and truncated at the 21st bp from the left and the 240th bp from the right. DADA2 eliminates low-quality sequences where there are ambiguous nucleotides. It identifies and eliminates chimeric sequences. It efficiently integrates the paired-read fusion process as part of its de-noising algorithm, which optimizes the quality of the complete amplicon sequences. The final ASV (amplicon sequence variant) table was rarefied to 3039 reads per sample for diversity analyses.

### 2.4. Diversity Indices

Qiime2 Alpha rarefaction curves were plotted to determine the adequacy of sequencing depth. Alpha diversity in wild and captive individuals was calculated using the Shannon, Simpson, and Pielou indices. The diversity among years (beta diversity) was estimated by the Bray–Curtis and Jaccard similarity indices and visualized by cluster non-metric multidimensional scaling (nMDS) and principal coordinate (PCO) analysis, and its statistical significance was estimated using SIMPROF in PRIMER v7 software. To assess and quantify the shared ASVs among wild and captive individuals, an UpSet plot was generated using the UpSetR package ver 1.4.0 [12] within the R statistical environment ver 4.3.1 [13].

### 2.5. Gut Microbiota Taxonomy of Wild and Captive E. morio

The taxonomic assignment of ASVs (amplicon sequence variants) was performed using Qiime2 with a silva_132_99_v3v4_q2_2019-7 pre-trained classifier (https://www.arb-silva.de/) accessed on 8 January 2024. The taxonomy abundance table was filtered to eliminate mitochondrial, chloroplast, and eukaryotic genomes, and relative abundance was calculated based on this taxonomic assignment. Taxonomy bar plots at the genus level were obtained in R [13]. A Venn diagram was created to visualize the microbiota shared by wild and captive individuals.

### 2.6. Metabolic Functional Prediction

To predict metabolic functions of the microbial communities, the Phylogenetic Investigation of Communities by the Reconstruction of Unobserved States PICRUSt2 (ver. 1.1.0) was used [14], with the NTSI cutoff of 2. The gut microbiota functions were predicted concerning the Kyoto Encyclopedia of Genes and Genomes (KEGG), KO (KEGG Orthology) Database [15].

### 2.7. Statistical Analysis

Alpha diversity: The Kruskal–Wallis test was used to determine statistical differences between years because the data did not meet the normality assumption determined by the Shapiro–Wilk test. The Mann–Whitney U test with Bonferroni adjustment was then used to determine which pairs of years showed significant differences. Metabolic functional analysis: Kruskal–Wallis tests were performed using XLSTAT 2025 to evaluate differences in functional expression across years. Pairwise comparisons were conducted using Dunn’s procedure with Bonferroni correction to identify statically significant interannual contrasts.

Beta diversity: Nonparametric multivariate analysis nMDS (non-metric multidimensional scaling) and PCoA based on Bray–Curtis and Jaccard similarity matrices were performed. A SIMPROF test (similarity profile) was used to assess graphical similarities between samples, using bacterial communities at the genus level [16,17]. Taxonomy: To assess differences in the gut microbial community structure of fish between groups (wild vs. captive) and over time, a permutational multivariate analysis of variance (PERMANOVA) [18] was applied using the PRIMER v7 package with a Bray–Curtis similarity matrix based on log(x + 1)-transformed abundance data. The design included two factors, group (wild vs. captive) as a fixed effect and year nested within group as a random effect, which allowed us to capture both overall differences by condition and progressive changes during adaptation to captivity. Ninety-nine permutations were used to estimate statistical significance. The SIMPER routine was applied to identify the taxonomic groups that most contributed to the dissimilarity between samples. Metabolic functional prediction: A Shapiro–Wilk normality test followed by a nonparametric multivariate analysis (nMDS) with SIMPROF to complement the clustering, as well as PCoA, were performed, and finally a Shade plot was constructed to visualize the gradient.

## 3. Results

### 3.1. Alpha Diversity of the Gut Microbiota of Wild and Captive E. morio

A total of 3040 amplicon sequence variants (ASVs) were obtained from 69 wild and captive *E. morio* samples. The mean frequency per sample was 13,510; min, 3040.0; max, 23,502.0. The rarefaction curve showed the number of observed ASVs in relation to sequencing depth. The plateau curves showed that the sampling effort was sufficient to capture the microbial diversity present in our samples. The gut microbiota of captive *E. morio* presented a higher number of observed ASVs than that of wild *E. morio.* Fewer ASVs were found in all 2024 captive individuals, indicating lower diversity than most wild individuals (Supplementary File S2).

The Shannon, Simpson and Pielou indices (alpha biodiversity) showed significant differences between sampling years, according to the Kruskal–Wallis test (H ≥ 203.64; *p* ≤ 0.000012).

**Figure 1:**
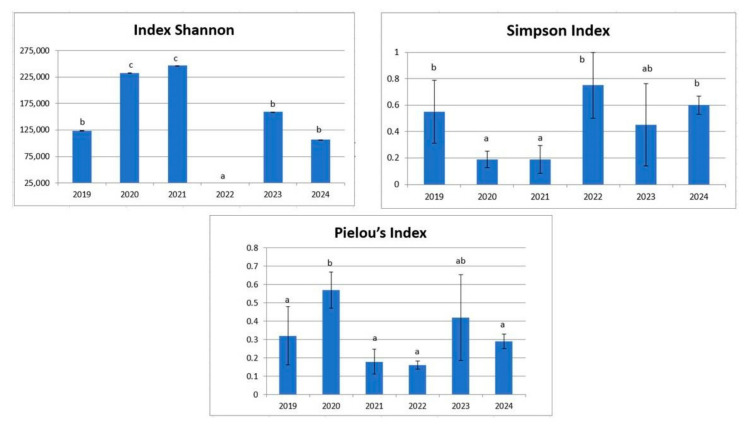
Alpha diversity indices in wild (2019) and captive (2020–2024) *E. morio* individuals using Kruskal–Wallis test to determine significant differences.

The diversity indices showed a significant change between the microbiota diversity of wild individuals (2019) and those kept in captivity for one year (2020) (Supplementary File S3). The gut microbiota of captive *E. morio* is higher than that of wild *E. morio*. The Shannon index showed very low values in 2022, which will be discussed later (Figure 1).

### 3.2. Beta Diversity Analysis of Wild and Captive E. morio Gut Microbiota

**Figure 2:**
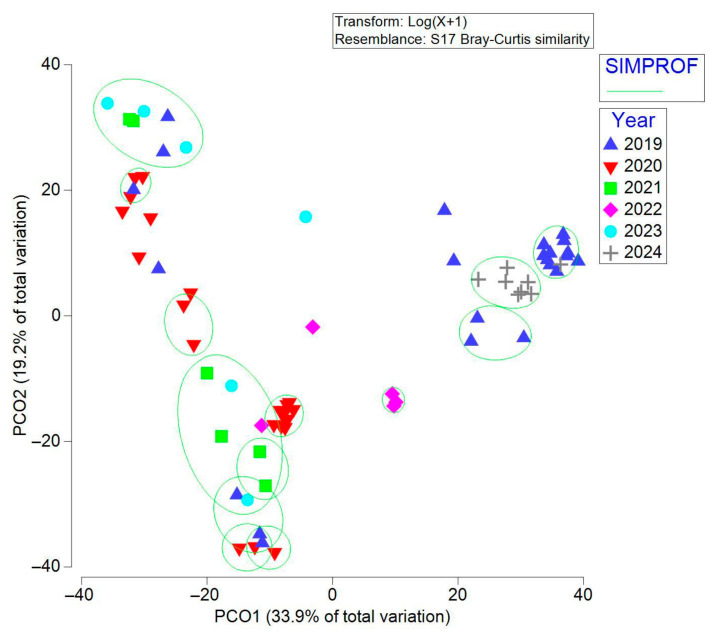
PCO analysis based on the Bray–Curtis and Jaccard similarity matrices calculated from ASV abundance data. Each point represents a sample, and proximity between points indicates similarity. Different colors indicate the sampling year. Wild individuals, 2019; captive individuals, 2020–2024.

The nMDS analysis based on the Bray–Curtis similarity matrix indicated that there was a higher similarity between the microbiota of wild individuals (2019) and those that have been in captivity for 5 years (2024), forming a cloud of points. The similarity based on the Jaccard coefficient did not show a defined pattern (Supplementary File S4). The PCoA based on the Bray–Curtis matrix explained 53.1% of the variation (33.9 % with component 1 and 19.2 % with component 2). We show that the similarity of ASVs is higher in wild individuals (2019) and those that have been in captivity for five years (2024) (Figure 2), coinciding with the clustering pattern of nMDS. The PCoA with the Jaccard matrix was not included because it explained less than 30 % of the variation.

**Figure 3:**
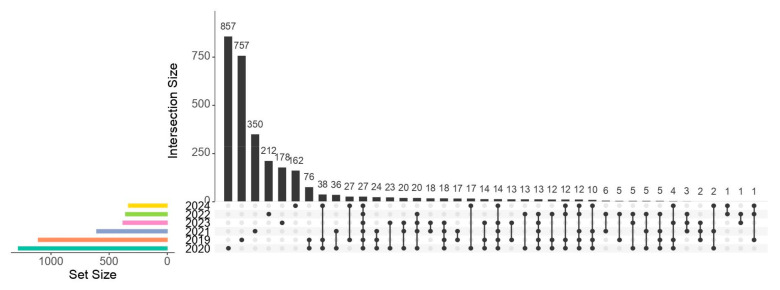
Unique or shared ASVs. The black vertical bar chart displays the intersection size (number of ASVs, with precise counts indicated above each bar) across *E. morio* sampling years; the dots below the bars indicate the intersecting years. The colored horizontal bar chart indicates the total number of ASVs (read counts) for each year.

Only 27 ASVs (0.88% of total ASVs, but with greater abundance: 70.8% of read counts) were shared across all sampling years for the 16S rRNA gene libraries, representing a small but highly abundant core of ASVs (Figure 3). The taxonomic assignment of some of these shared ASVs includes *Micrococcus* sp., *Acinetobacter* sp., *Staphylococcus* sp., *Vibrio* sp., *Bacillus* sp., *Shewanella* sp., *Photobacterium damselae, Pseudomonas* sp., and *Escherichia-Shigella* sp. (Supplementary File S5).

Most ASVs were unique to a single sampling year or shared across two or three sampling years (Figure 3). The highest number of unique ASVs was found in wild (2019) and captive (2020) individuals. Most wild (2019) and captive (2024) individuals, according to the Jaccard index, had high similarity, and are shown here to share 27 ASVs.

### 3.3. Changes in the Taxonomy of the Gut Microbiota of Wild E. morio During the Captivity Period

Twenty-nine phyla were identified in the gut microbiota of *E. morio* in captivity and nineteen in wild individuals. The ten phyla present exclusively in captive individuals are shown with shading (Supplementary File S6). The Venn diagram shows the number of phyla shared in the microbiota of wild and captive individuals (Supplementary File S7). The relative abundance of phyla per sample is presented in Supplementary File S8.

**Figure 4:**
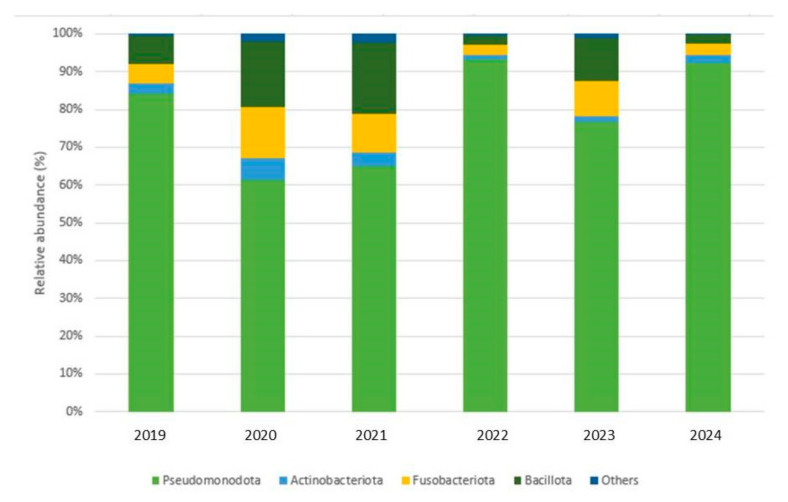
Relative abundance of predominant phyla in the gut microbiota of wild and captive *E. morio* (2019, wild individuals; 2020–2024, captive individuals).

Four dominant phyla were identified, while the rest were present in low relative abundance (<1%). Pseudomonadota was the predominant phylum followed by Bacillota, Fusobacteriota, and Actinomycetota. This pattern was consistent throughout the entire sampling period in both wild and captive individuals (Figure 4).

PERMANOVA analysis revealed no significant differences in the overall gut microbial community structure between wild (2019) and captive fish (2020) (Pseudo-F = 0.641, *p* = 0.565). However, the year nested within-group factor did show a highly significant effect (Pseudo-F = 6.13, *p* = 0.001), indicating that temporal variations explain much of the changes in microbiota (Supplementary File S9).

**Figure 5:**
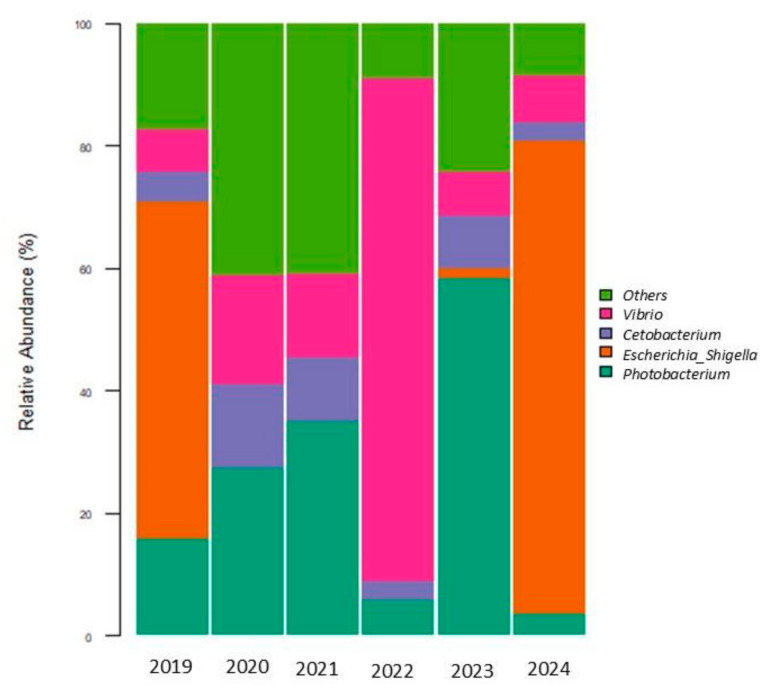
Relative abundance of predominant genera in *E. morio* gut microbiota. (2019, wild individuals; 2020–2024, captive individuals).

At the genus level, four genera were the most abundant in wild and captive individuals: *Photobacterium*, *Vibrio*, *Cetobacterium,* and *Escherichia-Shigella* (Figure 5). The relative abundances of these genera by year are shown in Supplementary File S10. Taxonomy results from Qiime2 showed the genera *Escherichia* and *Shigella* in the same group (*Escherichia-Shigella*). Other genera were found in low relative abundances (<3%) in the gut microbiota of *E. morio* in wild and captive individuals (Supplementary File S11).

Comparing the microbiota of wild (2019) and captive (2020) fish, the genera *Cetobacterium* sp., *Lactococcus* sp., and *Clostridium* sp. explained 65 % of the dissimilarity between groups. *Cetobacterium* sp. had the greatest difference in abundance, indicating a consistent contribution to group separation.

The gut microbiota showed significant changes after the transition from the wild state (2019) to captive conditions (2020–2023), evidenced by dissimilarities greater than 60 %. However, multivariate analysis (PCO and nMDS) revealed that in 2024, the community structure again approached the original profile. This apparent reconvergence suggests an adaptive and stabilizing process, in which some microbial characteristics of the wild state were partially recovered after several years in captivity.

Analysis of beta diversity based on the Jaccard index confirmed that the qualitative structure of the gut microbiota also underwent an abrupt change from the wild to captivity, with a pattern of partial reconvergence in 2024.

### 3.4. Functional Predictions of Gut Microbiota of E. morio

**Figure 6:**
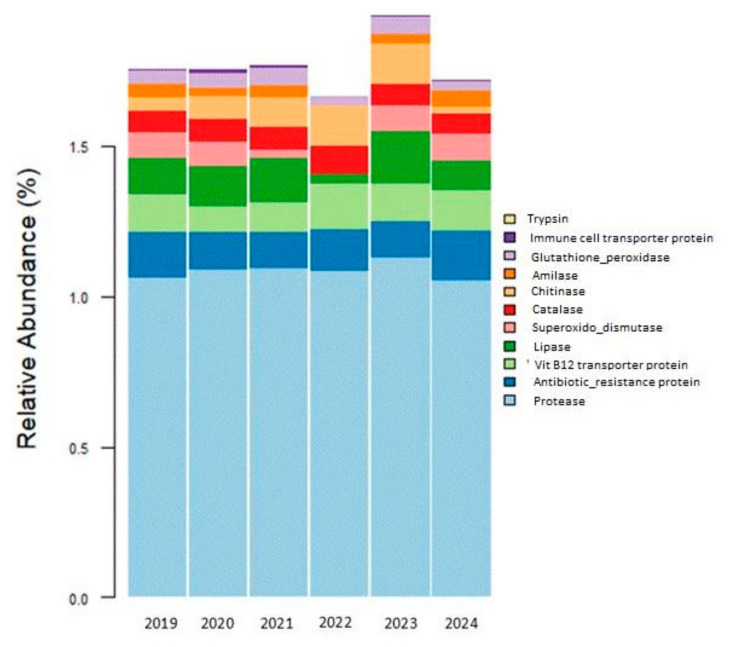
Relative abundance of functions of gut microbiota in *E. morio*, based on the classification of PICRUSt from KEGG Orthology (KO) database (2019, wild individuals; 2020–2024, captivity period).

Of all the functions that could be predicted (Supplementary File S12), ninety-eight percent of those identified were related to cellular maintenance, so we focused on the remaining 2% related to probiotic functions (Supplementary File S13). Predictive analysis of bacterial functions indicated that the most significant contribution of the microbiota to the host was related to digestive enzymes such as proteases, lipases, chitinases, and amylases. Antibiotic resistance and vitamin B12 transport were also recurrently detected. Other less frequent functions were directly associated with antioxidant enzymes (superoxide dismutase, catalase, and glutathione peroxidase) and the immune system. The codes (EC) and relative abundances of the enzymes are shown in Supplementary File S14. Although the microbiota in captive individuals is more diverse than in wild individuals, the metabolic functions mentioned above are maintained in both wild and captive individuals (Figure 6).

The nMDS multivariate analysis for metabolic predictions showed a stress value of 0.13, considered statistically acceptable, i.e., a reliable representation of the 2D distances. Distinct clustering by year was observed, especially 2019 and 2024. Captive fish samples from 2020, 2021, and 2023 showed greater dispersion (Supplementary File S15). The PCO results explained 70.5% of the variation (PCO 1, 50.1% and PCO 2, 20.4%), and the clustering was consistent with the nMDS, showing greater clustering between 2019 and 2024 and a greater range of dispersion in the remaining years (Supplementary File S16). In both groups, the 2022 samples were the most distant.

**Figure 7:**
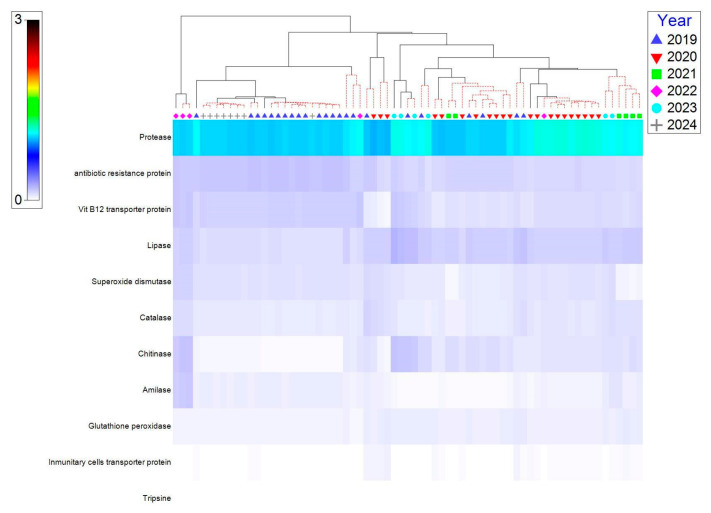
Shade map showing the relative abundance of metabolic functions in different wild (2019) and captive (2020–2024) individuals. The *x*-axis shows individuals grouped by year and the *y*-axis shows the identified metabolic functions. The color intensity indicates the relative abundance of each function: darker squares represent higher values, while lighter squares indicate lower abundances. The clusters in red indicate statistical associations by SIPROF.

Predicted metabolic functions were most similar in 2019 and 2024, as shown in the heat map (Figure 7). Functional abundances in 2022 showed the greatest difference between each other (this supports the nMDS and PCO results). The protease, lipase, catalase, superoxide dismutase, and antibiotic resistance proteins show higher intensities in later years, particularly in 2023–2024. Digestive functions (amylase, lipase, trypsin) are present, but some with low relative intensity in the early years. Functions related to oxidative stress and immunity (superoxide dismutase, catalase, glutathione peroxidase) appear to increase as captivity progresses.

## 4. Discussion

### 4.1. Diversity Findings

Here, for the first time, we described the changes in gut microbial communities in wild *E. morio* that were kept in captivity. The observed ASVs were higher in captive individuals compared to the wild ones. The Shannon diversity index showed a drastic decrease in 2022 (Figure 1). This may be because this index combines two key components of diversity, species richness and evenness. In this particular year, an increase in the relative abundance of the genus *Vibrio* was observed (Figure 5), along with a lower presence of other genera and also a decrease in evenness due to the predominance of *Vibrio* sp. The Simpson index indicates the probability that two individuals taken at random belong to the same taxon, with lower values representing greater diversity, as in 2021–2022 compared to wild individuals (2019). The Pielou index also measures the evenness in taxa abundances. 2022 showed the lowest values for this index, likely due to the predominance of *Vibrio* sp. Overall, these indices indicate greater diversity in captive individuals than in wild ones.

Our results contrast with another report [7], where the gut microbiota of wild and captive *Epinephelus akaara* (red-spotted grouper) was studied. The authors found that the alpha biodiversity of wild *E. akaara* was higher than that of captive *E. akaara.* They further comment that this pattern has been seen in other vertebrates, probably because wild animals, unlike those found in captivity, are exposed to different environmental conditions, including a greater variety of movement, seasonality, social interactions, and a wide variety of diets. However, it has been reported in other vertebrates that there are no differences in α-diversity between wild and captive individuals, as in lizards [19], even-toed ungulates (*Cetartiodactyla*), and two myrmecophagous species [20]. Although α-diversity was higher in wild individuals, the network complexity was greater in captive individuals due to the presence of bacteria with stronger positive correlations, which promotes cooperation and complementarity [7]. Still, our discrepancies with the results of *E. akaara* could be explained as a species-specific issue; there are contradictory ideas related to the change in the gut microbiota associated with food and other environmental factors [9]. In this sense, in a study that analyzed the gut microbiota of the largemouth bronze gudgeon (*Coreius guichenoti*) in the wild and in captivity, the results showed that captive fish had higher alpha diversity (measured by the Shannon index) compared to wild fish. This was attributed to the artificial feeding and environmental conditions in captivity, which allowed for the colonization of a wider range of bacteria [21]. This could also be the case for *E. morio*, but further studies are needed to corroborate this idea.

The similarity of the microbial community composition between wild individuals (2019) and those that have been in captivity for a longer period (2024), according to the Bray–Curtis index, is high. However, two groups can still be distinguished (Figure 2). Since this index considers both presence and abundance, this suggests that they share ASVs, but the abundance of these ASVs differs.

PERMANOVA showed that time (years) is a determining factor in the modification of the intestinal microbiota in fish, but not the initial change in environment, from wild to captivity (2019–2020). Microbiological adaptation is progressive and not immediate. This analysis suggests that the microbial structure varies significantly with time, probably due to physiological adaptation, changes in diet, or captive conditions.

### 4.2. Core Microbiota

The unique and shared ASVs (UpSet plot, Figure 3) indicate that individuals, whether wild or captive, tended to exhibit a higher proportion of unique (and low-abundance) ASVs and a small portion of highly abundant ASVs shared between all years that formed a core microbiota. The taxonomic assignment of these shared ASVs coincided with several of the genera isolated from the gut contents of *E. morio* in our previous study on culturable gut microbiota [22]. Concerning taxonomic assignments associated with the 27 ASVs forming the core microbiota, we found that these ASVs were classified as *Micrococcus* sp., *Bacillus* sp., *Acinetobacter* sp., *Staphylococcus* sp., *Vibrio* sp., *Pseudomonas* sp., and *Escherichia-Shigella* sp., among others (Supplementary File S5). At the same time, we gathered some significant data concerning each. The presence of *Micrococcus* sp. has been reported in other fish and could have potential as a probiotic in *E. morio* due to its proteolytic and amylolytic activity [22]. It has been shown to stimulate growth and the immune system in tilapia [23]. The probiotic capacity of *Bacillus* sp. is well known; it produces digestive enzymes such as lipases and proteases. It improves the feed conversion ratio, which helps improve growth. In addition, *Bacillus* competes with pathogenic bacteria for nutrients and space [24]. *Acinetobacter* sp. is a bacterium that has been reported as a pathogen in fish. Infected fish presented lesions in organs such as the liver, kidney, spleen, and heart. Some strains of *Acinetobacter* sp. have shown beneficial effects as probiotics and have been shown to significantly improve growth, immune activity, and resistance to diseases such as those caused by *Aeromonas* [25].

*Staphylococcus* sp. is a pathogen capable of producing enterotoxins resistant to adverse conditions. Some species such as *Staphylococcus aureus* are not part of the natural microbiota of wild fish, suggesting that their presence is the result of external contamination, mainly of human origin. These bacteria can colonize fish during the capture and transportation stages [26]. *Vibrio* sp. is an opportunistic pathogen that increases in abundance when there are stressful conditions or in the presence of parasites. It usually causes a decrease in diversity by competitive exclusion with other bacteria, influencing the health and microbial balance of the host. A study has determined that in zebrafish larvae (Danio rerio), *V. cholerae* can significantly displace *Aeromonas veronii* [27]. *Pseudomonas* sp. and *Escherichia-Shigella* sp. are genera associated with water contamination. The detection of these genera as an abundant core shared within wild and captive fish could indicate possible fecal contamination of water or feed. But on the other hand, these genera could have a significant functional role, as we will discuss further.

### 4.3. Gut Microbiota Taxonomy of Wild and Captive E. morio

The gut microbial community of *E. morio* is characterized by a predominance of the phylum Pseudomonadota, followed by Bacillota, Fusobacteriota, and Actinomycetota, in both wild and captive *E. morio*. These results are consistent (also at the genus level) with those that we previously obtained when we studied the cultivable gut microbiota of *E. morio* (22). Recent studies highlight that these four phyla, along with, Bacteroidota and Tenericutes, are the most dominant in the gastrointestinal tract of teleost fish [1,28,29].

The taxonomic diversity found in this study indicates that captive individuals exhibited a greater number of phyla compared to wild individuals, as shown in Supplementary File S6, where 10 phyla were exclusive of captive individuals, such as *Abditibacteriota*, *Gemmatimonadota*, *Halanaerobiaeota*, *Latescibacterota*, *Methylomirabilota*, *Nitrospirota*, and *Thermotogota*. Its presence might be related to the adaptation of host fish to captivity conditions and diet changes, but more specific studies would be needed to demonstrate this hypothesis.

Our results agree with a report on *Epinephelus akaara* [7], where both wild and captive fish exhibited a lower abundance of Bacillota and Bacteroidota compared to Pseudomonadota. The relative abundance of Pseudomonadota in wild and captive individuals suggests that it plays a primary role as the main component of the core gut microbiota of marine fish and particularly of *E. morio*. Similar results were obtained in *Epinephelus coioides* [30], where it was determined that Pseudomonadota, Bacillota, and Actinomycetota were the most abundant bacterial phyla, and a high relative abundance of Pseudomonadota in the hindgut was also reported for wild and captive *Tor tambroides*, Malaysia’s most valued freshwater fish species [8]. Some studies have proposed that the predominance of Pseudomonadota or Bacillota has been directly associated with diet composition. These microorganisms are well adapted to the conditions of the fish guts and the aquatic environment [28,31].

The genera *Photobacterium*, *Cetobacterium*, *Vibrio*, and *Eschericha-Shigella* were predominant in both wild and captive individuals. In wild fish, the relative abundance of *Escherichia-Shigella* is higher, followed by *Vibrio*, *Photobacterium*, and *Cetobacterium*. During the first four years of captivity, there was a drastic decrease in *Escherichia-Shigella* and the predominance of *Photobacterium*. Some studies suggest that *Photobacterium* can contribute to digestion as well as the supply of fatty acids and vitamins [32,33]. Its association with fish may be symbiotic, growing within fish for the formation of light organs, or as decomposers of dead fish, or as an agent of disease [34]. Some of the 15 known species of *Photobacterium* have evolved into pathogens of marine life as *Photobacterium damselae* [35]. Some of these diseases affect commercially important fish and can therefore indirectly impact human health through their consumption. The presence of *Photobacterium* sp. and *Vibrio* sp. is related to the type of diet consumed, and it is commonly found in carnivorous fish such as Atlantic salmon (*Salmo salar*) and Black rockcod, among others [1].

The increase in the relative abundance of the genus *Vibrio* in captive individuals in 2022 could be explained by a parasite infection. Fish showed changes in behavior this year, such as rubbing their bodies in the pond. Due to this, a skin smear was performed, and some protozoa were found. To treat the infection, the fish were treated with a formaldehyde bath and fresh water. Bacteria of the *Vibrio* genus are opportunistic pathogens; it is possible that a parasite infection could generate stress, and in such a scenario, the abundance of *Vibrio* increased. The relative abundance of the other genera in 2022 decreased, probably due to interspecific competition with *Vibrio* sp.

In our study, we found a high percentage of *Escherichia-Shigella* in wild individuals (2019) and in individuals who had spent five years in captivity (2024). The taxonomy results generated by Qiime2 showed the genera *Escherichia* and *Shigella* in the same group (*Escherichia*-*Shigella*) (Figure 5), due to their closely phylogenetic relationship [36], so we could not distinguish them independently. *Escherichia* and *Shigella* are enterobacteria typically associated with the intestines of humans and terrestrial animals. Their detection in marine fish is often an indicator of fecal contamination in water (rivers, estuaries, coastal areas).

### 4.4. Functional Implications

PICRUSt2 is a computational approach used to predict the metabolic functions of the microbiota from 16S rRNA sequencing data, and it has important advantages and limitations to consider. Some of its strengths include that (1) it allows the prediction of metabolic functions using only 16S rRNA data, which is much more affordable than shotgun metagenomics, (2) it uses databases such as KEGG Orthologs (KO), MetaCyc, and EC numbers, enabling detailed functional predictions, (3) it incorporates evolutionary models to place sequences into more robust reference phylogenetic trees, (4) it works with ASVs, making it more accurate, and (5) it is a well-documented and widely tested tool, with strong support in the scientific literature. Some of its limitations are that (1) it does not directly measure functions; it only predicts them based on phylogeny and known functions of reference genomes, (2) for organisms that are poorly represented in genomic databases, predictions may be unreliable or absent, (3) metabolic functions can vary significantly among closely related strains due to horizontal gene transfer or other factors, and (4) it cannot detect expressed or regulated functions because it does not account for whether a gene is active, silenced, or regulated in a given context [37]. Despite these limitations, it is an excellent tool when a quick, cost-effective, and general overview of the functional potential of the microbiota based on 16S rRNA is desired. For more precise or actual functional studies, methods such as shotgun metagenomics or metatranscriptomics would be required [37]. We employed 16S rRNA gene metabarcoding for a first approximation to metabolic predictions when there was little if any information available, as in the case of *E. morio.* Our taxonomic results and some metabolic predictions agree with our previous study with isolates of the intestinal microbiota of *E. morio*, demonstrating the effectiveness of this approach [22].

Among the most abundant genera, *Photobacterium* sp., *Vibrio* sp., and *Escherichia-Shigella* sp. are considered pathogens, as well as others with lower relative abundance such as *Staphylococcus* sp. and *Clostridium* sp. On the other hand, in the microbiota of *E. morio*, there are other bacteria with probiotic potential such as *Lactobacillus* sp., *Bacillus* sp., *Lactococcus* sp., *Streptococcus* sp., *Weissella* sp., and *Turicibacter* sp. Despite the presence of these pathogenic bacteria, there was no evidence of disease in the fish, indicating that their immune systems were able to cope with these bacteria. The presence of bacteria with probiotic potential supports the predicted functions of the *E. morio* microbiota.

The predictive analysis of bacterial functions indicates that the main contribution of the microbiota to the host seemed to be related to digestive enzymes such as proteases, lipases, chitinases, and amylases. Antibiotic resistance and vitamin B12 transporters were other abundant functions (Figure 6). These functional categories showed statically significant differences between years, as confirmed by Kruskal–Wallis tests followed by Dunn’s pairwise comparisons with Bonferroni correction (Supplementary File S17). The primary metabolic function that was attributed to the *E. morio* microbiota was proteolytic activity. Proteinases are polyfunctional enzymes catalyzing the hydrolytic degradation of proteins [38]. *E. morio* is a carnivorous species, so its diet has a high protein component. In the wild, it consists of smaller fish, crustaceans, cephalopods, etc., and in captivity, the diet was composed of a mixture of dry foods such as fish meal and fresh food such as squid, fish, and shrimp. This justifies the high relative abundance of proteases found in the metabolic predictions in the gut microbiota in both wild and captive individuals.

The proteolytic activity of the gut microbiota has also been reported in other marine fish. In a study carried out on individuals of the genus *Centropomus*, strains were cultured and isolated from the intestinal tract, and it was found that some of them could produce extracellular proteases [33].

Another function that was attributed to the *E.morio* microbiota was amylolytic activity. In the wild, carnivorous fish such as *E. morio* do not directly consume processed carbohydrates but can ingest naturally complex carbohydrates by feeding on prey containing these substances such as benthic crustaceans containing glycogen and chitin [39,40]. In captive individuals, there are certain elements of diet that contain carbohydrates, such as flour, so these bacteria may be contributing to the degradation of these diet components.

According to a previous study performed by our team [22], bacterial isolates from different genera (*Micrococcus* sp., *Bacillus* sp., *Photobacterium damselae*, *Staphylococcus* sp., *Enterococcus* sp., *Shewanella* sp., and *Vibrio* sp.) obtained from the gut microbiota of *E. morio*, presented proteolytic activity, and two other genera (*Micrococcus* sp. and *Bacillus* sp.) showed amylolytic activity [22]. It is worth noting that all these isolates belong to genera that form the core microbiota (Supplementary File S5). The isolates will be further characterized for acid pH tolerance, antibiotic resistance, bile salt resistance, antagonism, etc. The long-term objective is to select isolates with probiotic potential to design a bacterial consortium that can be included in the feed of captive groupers to improve nutrition.

One of the functions of probiotics is the production of vitamins that aid the host’s metabolism. Regarding the synthesis of vitamin B12, our results indicate that this could be one of the probiotic functions that the *E. morio* microbiota provides to the host. These results are consistent with a recent study that demonstrated that the anaerobic bacterium *Cetobacterium somerae* (the *Cetobacterium* genus was also detected in *E. morio*), common in the gut of freshwater fish, produces vitamin B12. This production improves host resistance to pathogenic infections by strengthening interactions within the gut microbiota and the tight junctions of the intestinal barrier. The researchers concluded that the vitamin B12 synthesized by this bacterium plays a key role as an immune modulator in fish [41].

Antibiotic resistance is another function that could be attributed to the microbiota of *E. morio*. Bacteria of the genus *Aeromonas* have been reported to be involved in antibiotic resistance. One example is *Aeromonas veronii*. This species has been identified in the gut of the goldfish (*Tor putitora*), a Himalayan freshwater fish. *A. veronii* has been shown to harbor multiple antibiotic resistance genes, including those encoding β-lactamases, as well as multidrug resistance genes. According to the authors, this may suggest a potential risk of dissemination to other bacteria due to horizontal gene transfer [42]. In our study, the *Aeromonas* genus was identified in both wild and captive individuals. This could be related to the identification of antibiotic resistance as one of the possible functions of the *E. morio* microbiota.

Among the possible functions of the intestinal microbiota of *E. morio*, its relationship with antioxidant enzymes was also predicted. Some bacteria present in the intestinal microbiota of fish are associated with the modulation of antioxidant enzymes, playing a crucial role in protection against oxidative stress. The genera *Lactobacillus*, *Cetobacterium*, and *Bacillus* have been linked to antioxidant capacity in fish [43]. These genera were also identified in our study as part of the *E. morio* microbiota.

The presence of *Lactobacillus* in common carp (*Cyprinus carpio*) is associated with higher levels of reduced glutathione (GSH) and a decrease in the activity of enzymes such as glutathione peroxidase (GPx) and glutathione reductase (GR), indicating a reduction in oxidative stress [43].

In statistical analysis, while some predicted metabolic functions were maintained across years, several others (such as amylase, lipase, chitinase, glutathione peroxidase, antibiotic resistance, and vitamin B12 transport) exhibited significant interannual variation, as demonstrated by nonparametric Kruskal–Wallis and Dunn–Bonferroni tests.

The greater dispersion of data in 2020, 2021, and 2023 may indicate functional variability due to transitional conditions or environmental fluctuations. The differentiation in the functional profile over time, statistically supported by Kruskal–Wallis and Dunn–Bonferroni tests, reinforces the hypothesis of a progressive functional adaptation to captive conditions. The shared maps reinforce the hypothesis that there is an increase in adaptive functions in response to an artificial diet and captive conditions.

### 4.5. Environmental/Pollution Impact

Under aquaculture conditions, fish can be exposed to bacteria of human or animal origin through contaminated feed or poor management of water systems [1]. *Escherichia coli* and *Shigella sonnei* were also isolated from the majority of four fish species in a study conducted on the Ayuquila River in western Mexico [44], concluding that Enterobacteriaceae dominate the bacterial microbiota of both water and fish in the river, suggesting a significant influence of human activities. In accordance with this idea, wild *E. morio* individuals were collected in 2019 from the northern coast of Yucatán, where incidents of coastal pollution have been reported. A study conducted by the Federal Commission for the Protection against Sanitary Risks [45] at 24 locations along the Yucatan coast revealed an increase in the presence of bacteria and protozoa, mainly from untreated sewage. Although contamination levels are still considered suitable for recreational use according to World Health Organization (WHO) standards, the increase indicates a worrying trend.

On the other hand, the presence of the *Escherichia-Shigella* genera in the microbiota of captive individuals (2024) may be due to a spike in coastal pollution that affected the water in the reservoir that supplies the experimental ponds. Another epidemiological study conducted in 2024 indicated that *Enterococcus* levels close to the permissible limit were recorded on the Yucatan coast [46]. Similarly to 2019, water pollution could be the cause of the presence of these pathogenic bacteria and could also be the cause of the decline in microbial diversity, regardless of whether the individuals are wild or in captivity.

## 5. Conclusions

Genetic diversity was higher in captive individuals compared to wild individuals based on alpha diversity indices. Wild and captive individuals shared both ASVs and taxonomic groups, which supports the hypothesis of a core microbiota in *E. morio*. The change in microbiota composition is affected more by time in captivity than by the transition from the wild to captivity, which is evidence that the microbial structure varies significantly over time, probably due to physiological adaptation, changes in diet, or captive conditions. The microbiota of *E. morio* is structured with a few taxonomic groups of high relative abundance present in wild and captive individuals and many groups with low relative abundance. On the other hand, 300 genera were identified exclusively in captive individuals, which probably began to be established due to the adaptation of individuals to the conditions of captivity. The similarity in taxonomy and abundance of the gut microbiota between wild individuals (2019) and those after four years in captivity (2024) could be related to contamination events that increased the presence of *Escherichia-Shigella* and decreased diversity in both experimental groups. The gut microbiota in *E. morio* appears to be involved in metabolic processes related to food digestion and absorption, vitamin B12 transport, antibiotic resistance, and oxidative stress. These functions are likely performed by the core microbiota of *E. morio*, and their variation over time is statistically supported by Kruskal–Wallis and Dunn–Bonferroni tests, confirming functional shifts associated with captivity. The differentiation in functional profile over time supports the idea of progressive functional adaptation under captive conditions. We can conclude that in *E. morio*, the microbiota established in captivity conditions is sensitive to environmental changes, such as water pollution, which can cause a decrease in diversity. The proposed hypothesis is confirmed by identifying a core microbiota in the intestinal content of *E. morio* and the presence of 10 exclusive phyla in captive individuals and the predicted functions associated with digestion and stress in captive conditions. This study contributes to the knowledge of the microbiota of marine fish and its possible functions. It can be taken as the main background for more exhaustive studies on the gut microbiota in *E. morio*, using a metagenomic approach.

## Data Availability

The original contributions presented in this study are included in the article/Supplementary Materials. Further inquiries can be directed to the corresponding author.

## Supplementary Materials

The following supporting information can be downloaded at: https://www.mdpi.com/article/10.3390/microorganisms13081792/s1, File S1: Primer used in the illumina sequencing of the V3–V4 region of rRNA16s in red grouper intestinal contents; File S2: Observed ASVs as a function of sampling depth in gut microbiota of wild and captive *E. morio*; File S3: Alpha diversity indices of gut microbiota in wild and captive individuals of *E. morio*; File S4: nMDS beta diversity Bray-Curtis and Jaccard; File S5: Relative abundance of ASVs shared in gut microbiota of wild and captive individual of *E. morio*; File S6: Phyla in gut microbiota of *E. morio* in wild and captive individuals; File S7: Venn diagram; File S8: Relative abundance of Phylum in gut microbiota of *E. morio*; File S9: PERMANOVA statistics of Beta diversity; File S10: Relative abundance of main genus in gut microbiota of *E. morio*; File S11: Genera in low relative abundance (<3%) of gut microbiota of wild and captive *Epinephelus morio*; File S12: Relative abundance of functional predictions; File S13: Probiotic function of interest; File S14: Code EC and abundance enzyme; File S15: nMDS predictions functions; File S16: PCO predictions functions; File S17: Statistical analysis (Kruskal Wallis) for metabolic predictions.
