# Supplementary material for: Composition and Structure of Gut Microbiota of Wild and Captive Epinephelus morio via 16S rRNA Analysis and Functional Prediction
Source: Microorganisms. 2025 Jul 31;13(8):1792. doi: 10.3390/microorganisms13081792 (PMC12388694; doi:10.3390/microorganisms13081792)
Supplement: Supplementary file 1 [file microorganisms-13-01792-s001.zip › File S1 Primers Illumina.pdf]

| File S1. Primer used in the illumina sequencing of the V3-V4 region of rRNA16s in red grouper intestinal contents. | |
|---|---|
| Forward Primer | 5'TCGTCGGCAGCGTCAGATGTGTATAAGAGACAGCCTACGGGNGGCWGCAG |
| Reverse Primer | 5'GTCTCGTGGGCTCGGAGATGTGTATAAGAGACAGGACTACHVGGGTATCTAATCC |
