# Supplementary material for: Composition and Structure of Gut Microbiota of Wild and Captive Epinephelus morio via 16S rRNA Analysis and Functional Prediction
Source: Microorganisms. 2025 Jul 31;13(8):1792. doi: 10.3390/microorganisms13081792 (PMC12388694; doi:10.3390/microorganisms13081792)
Supplement: Supplementary file 1 [file microorganisms-13-01792-s001.zip › File S10. Relative abundance genus.pdf]

File S10. Relative abundance of main genus in gut microbiota of *E. morio*

	2019	2020	2021	2022	2023	2024	Mean	SD
Photobacterium	15.8461737	27.4730096	35.0444225	5.96906877	58.368981	3.56614018	24.377966	20.6085068
Escherichia_Shigella	54.9022133	0	0	0	1.49171877	77.1182955	22.2520379	34.6201981
Cetobacterium	5.05744166	13.5069493	10.2939564	2.79039158	8.71448942	3.20417901	7.26123456	4.2807276
Vibrio	6.71559581	17.8912236	13.7819458	82.2046726	7.11856971	7.46544916	22.5295761	29.5758736
Others	17.4785756	41.1288174	40.8796753	9.03586706	24.3062411	8.64593617	23.5791854	14.6938942
