# Supplementary material for: Composition and Structure of Gut Microbiota of Wild and Captive Epinephelus morio via 16S rRNA Analysis and Functional Prediction
Source: Microorganisms. 2025 Jul 31;13(8):1792. doi: 10.3390/microorganisms13081792 (PMC12388694; doi:10.3390/microorganisms13081792)
Supplement: Supplementary file 1 [file microorganisms-13-01792-s001.zip › File S3. Statistic Alpha diversity indices.pdf]

Alpha diversity indices of gut microbiota in wild and captive individuals of *E. morio.*

Year	S (total de taxones)	N	J' (Pielou's)	Shannon-Wiener	Simpson
2019	46,00000	100,00000	0,32261	1,23885	0,55469
2020	60,71429	100,00000	0,57460	2,32667	0,19639
2021	76,00000	100,00000	0,59500	2,46617	0,19585
2022	43,60000	100,00000	0,17793	0,74774	0,75442
2023	45,83333	100,00000	0,42433	1,59440	0,45150
2024	38,75000	100,00000	0,29155	1,06694	0,60603

Comparative summary of alpha diversity indices (Kuskal wallis).

Índice	Years	Statistic	p-valor
J'**(Pielou's)**	2019-2024	303.649	0.000012
**Shannon-Wiener '**	2019-2024	311.403	0.000009
**Simpson**	2019-2024	324.573	0.000005
