# Supplementary material for: Composition and Structure of Gut Microbiota of Wild and Captive Epinephelus morio via 16S rRNA Analysis and Functional Prediction
Source: Microorganisms. 2025 Jul 31;13(8):1792. doi: 10.3390/microorganisms13081792 (PMC12388694; doi:10.3390/microorganisms13081792)
Supplement: Supplementary file 1 [file microorganisms-13-01792-s001.zip › File S6. Phylum Wild and Captive.pdf]

File S6. Phyla in gut microbiota of *E. morio* in wild and captive individuals.

**Phylum**	**Wild**	**Captive**
Abditibacteriota	-	+
Acidobacteriota	+	+
Actinomycetota	+	+
Bacillota	+	+
Bacteroidota	+	+
Bdellovibrionota	+	+
Campylobacterota	+	+
Chloroflexota	+	+
Deinococcota	+	+
Desulfobacterota	+	+
Eremiobacterota (WPS-2)	+	+
Fusobacteriota	+	+
Gemmatimonadota	-	+
Halanaerobiaeota	-	+
Halobacterota	+	+
Latescibacterota	-	+
Methylomirabilota	-	+
Myxococcota	+	+
Nitrospirota	-	+
Patescibacteria	+	+
Planctomycetota	+	+
Pseudomonadota	+	+
SAR324_clade (Marine_group_B)	-	+
Spirochaetota	+	+
Sva0485	-	+
TA06	-	+
Thermotogota	-	+
Verrucomicrobiota	+	+
WPS-2	+	+
