# Supplementary material for: Composition and Structure of Gut Microbiota of Wild and Captive Epinephelus morio via 16S rRNA Analysis and Functional Prediction
Source: Microorganisms. 2025 Jul 31;13(8):1792. doi: 10.3390/microorganisms13081792 (PMC12388694; doi:10.3390/microorganisms13081792)
Supplement: Supplementary file 1 [file microorganisms-13-01792-s001.zip › File S9. PERMANOVA statistics of Beta diversity.pdf]

PERMANOVA statistics of Beta diversity.						
			Unique			
Source	df	SS	MS	Pseudo-F	P(perm)	perms
Gr	1	9154,7	9154,7	0,64147	0,565	337
Ye (Gr)	4	32240	8060,1	6,1279	0,00	999
Res	63	82865	1315,3			
Total	68	1,259E+05				

Grupo (Silvestre vs Cautiverio)

Pseudo-F = 0.6415, p = 0.565 (no significativo)

Año anidado en Grupo

Pseudo-F = 6.13, p = 0.001 (muy significativo)
