# Supplementary figures and images for: Composition and Structure of Gut Microbiota of Wild and Captive Epinephelus morio via 16S rRNA Analysis and Functional Prediction
Source: Microorganisms. 2025 Jul 31;13(8):1792. doi: 10.3390/microorganisms13081792 (PMC12388694; doi:10.3390/microorganisms13081792)

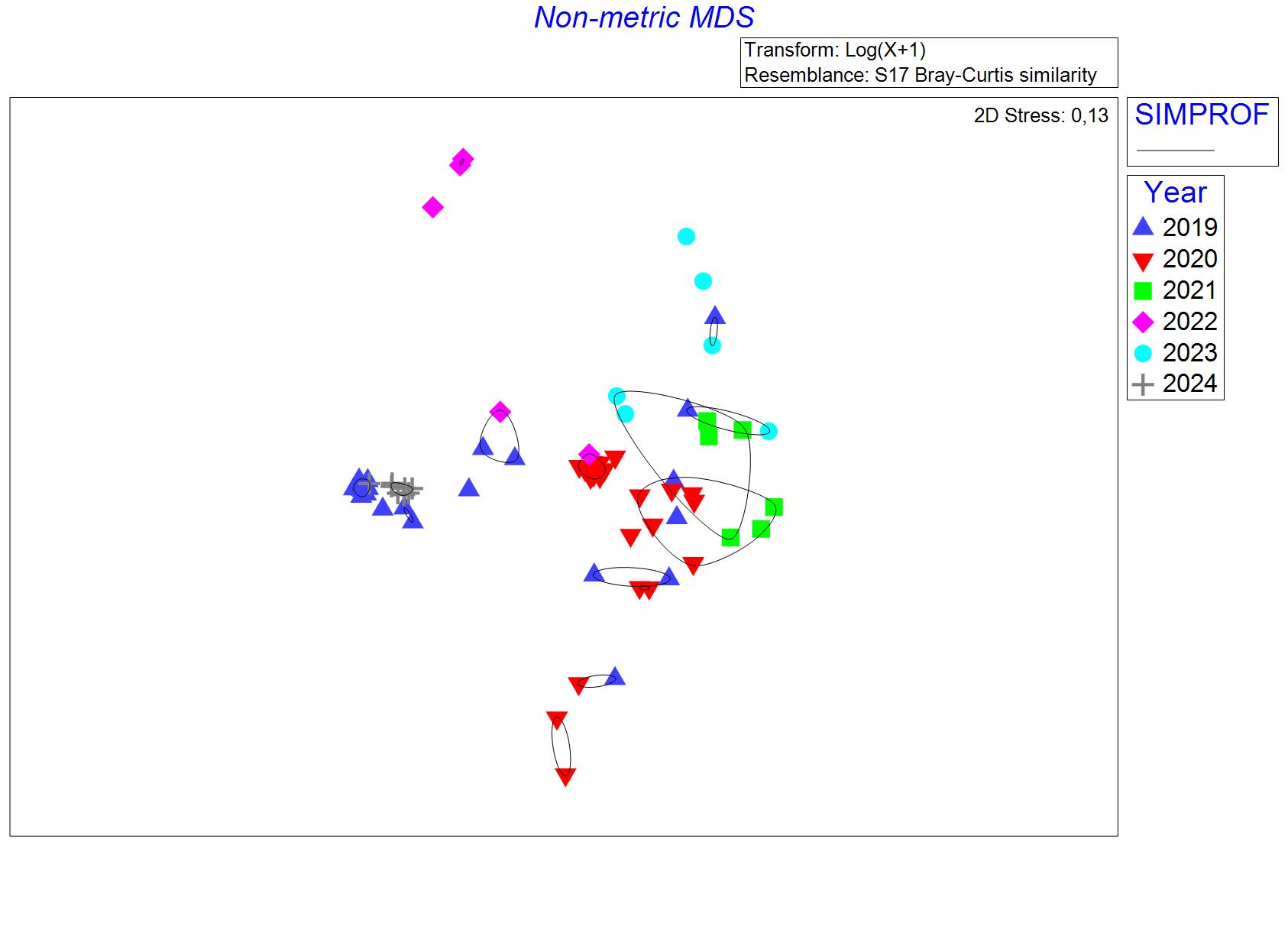

Supplement: Supplementary file 1 [file microorganisms-13-01792-s001.zip › File S15. nMDS Predictions functions.jpg]

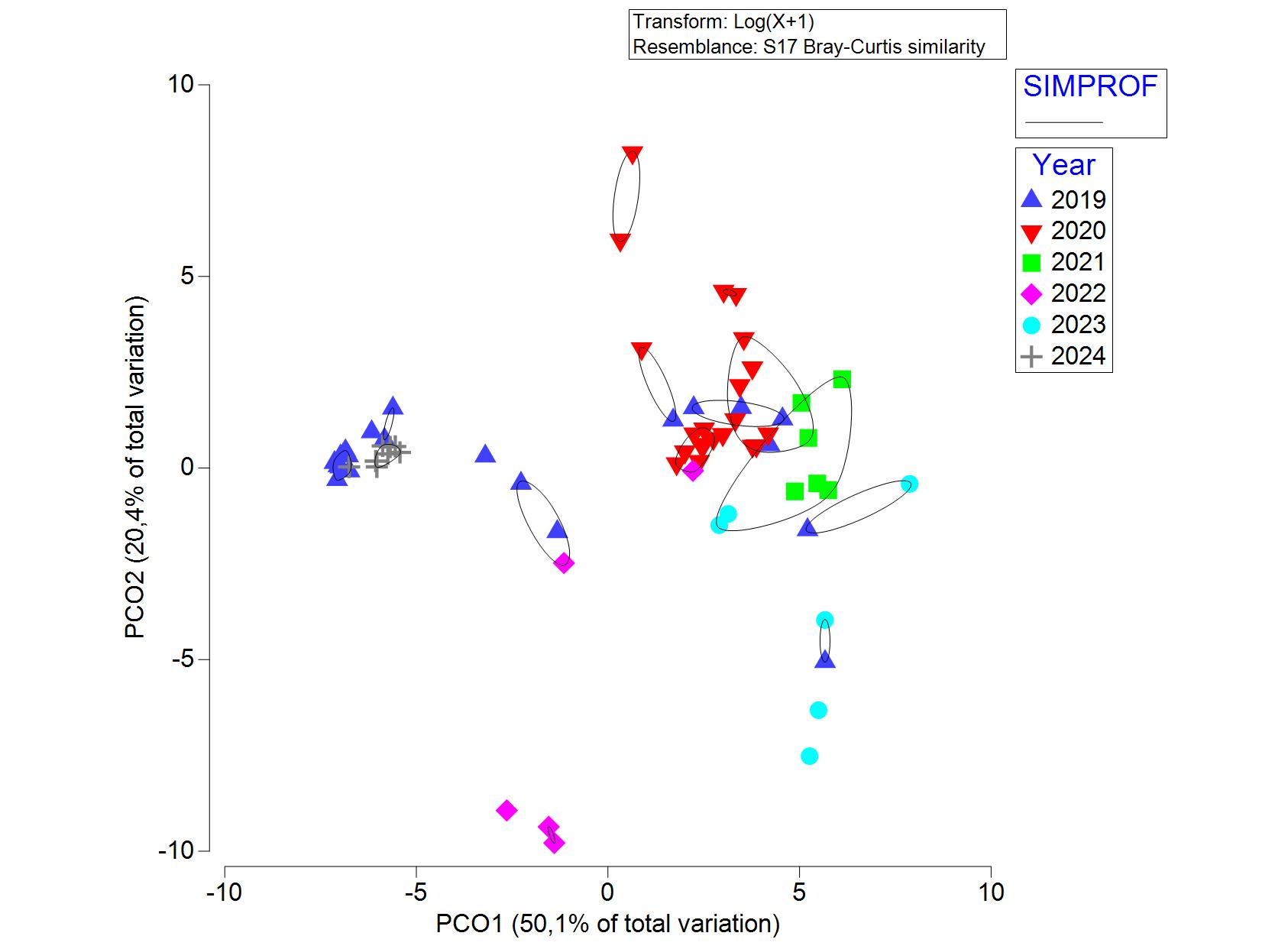

Supplement: Supplementary file 1 [file microorganisms-13-01792-s001.zip › File S16. PCO Predictions functions.jpg]

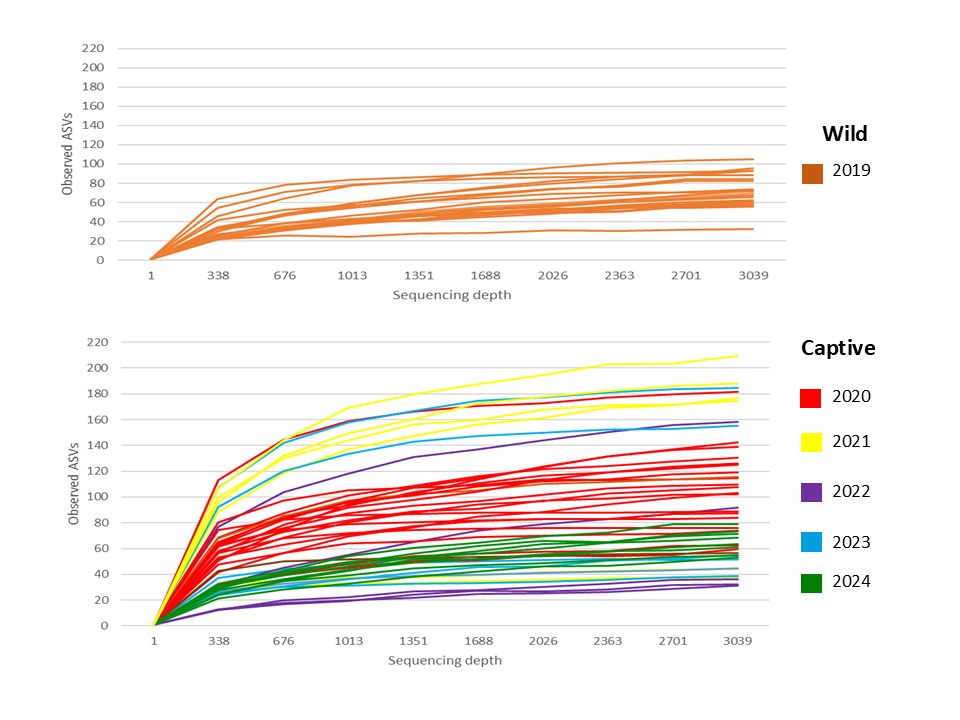

Supplement: Supplementary file 1 [file microorganisms-13-01792-s001.zip › File S2. Rarefacción.jpg]

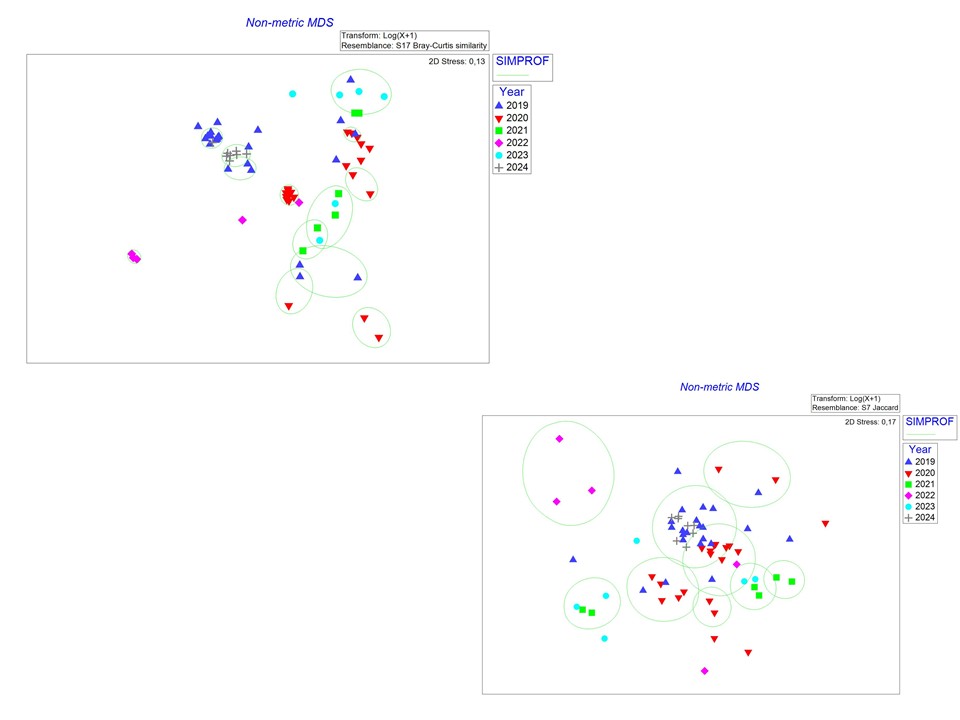

Supplement: Supplementary file 1 [file microorganisms-13-01792-s001.zip › File S4. nMDS Bry curtis-Jaccard.jpg]

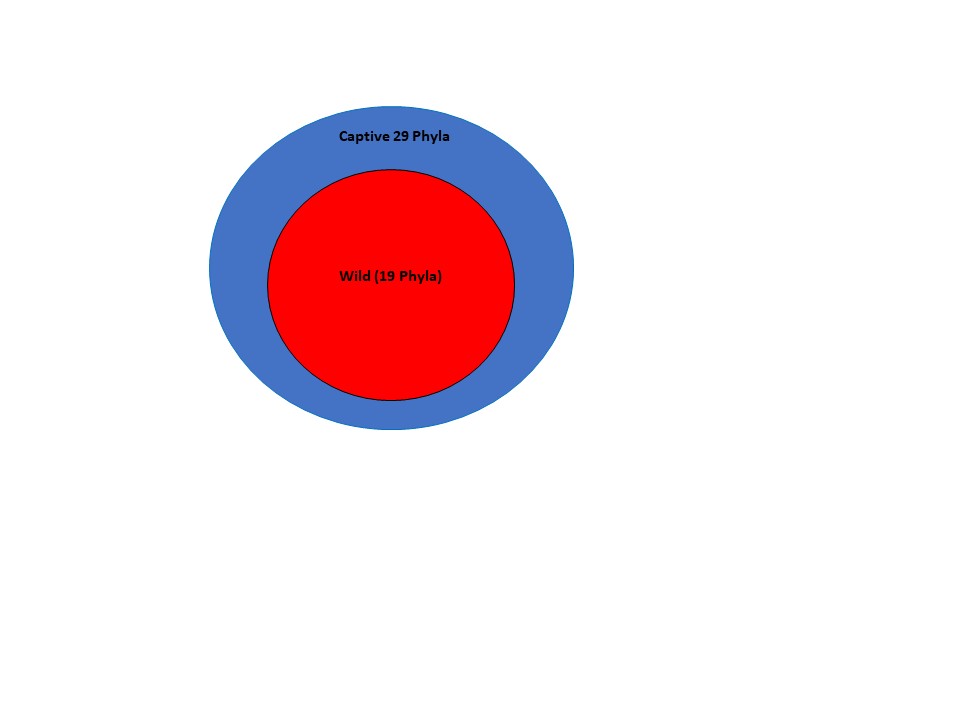

Supplement: Supplementary file 1 [file microorganisms-13-01792-s001.zip › File S7. Venn Diagram.jpg]
